# Androgen receptor gene polymorphism and biological age markers in men

**DOI:** 10.1038/s41598-025-22090-3

**Published:** 2025-10-31

**Authors:** Agnieszka Żelaźniewicz, Judyta Nowak-Kornicka, Adam Dąbrowski, Bogusław Pawłowski

**Affiliations:** 1 Department of Human Biology, University of Wrocław, Wrocław, Poland; 2 Laboratory of Molecular Diagnostics “Bio-Genetik” NZOZ, Wrocław, Poland

**Keywords:** Aging, Trade-offs, Receptor sensitivity, AR CAGn polymorphism, Klotho, DHEA/S, Predictive markers, Biological anthropology, Evolutionary developmental biology, Evolutionary biology

## Abstract

Inter-individual differences in aging rates may stem from the trade-offs between reproductive effort and somatic maintenance, with higher reproductive effort potentially reducing healthspan and lifespan. In men, these trade-offs are thought to be regulated by androgens, where high androgen levels incur biological costs. However, the relationship between androgen levels, health, and aging is complex, and the role of androgen receptor (AR) sensitivity in aging remains unclear. Thus, this study aimed to investigate whether genetic polymorphisms in the AR gene, related to receptor affinity and androgen sensitivity, predict biological age in men. The analyses included 131 healthy men (mean age: 35.4, age range 30–45). Biological age was assessed using physiological markers, including klotho, inflammatory markers, oxidative stress, total antioxidant capacity, and DHEA/S levels. AR sensitivity was estimated based on CAG repeat length in exon 1 of the AR gene (AR CAGn), with shorter repeats indicating higher sensitivity. The independent effect of testosterone (T) level, as well as its potential interaction with AR CAGn, was also verified. Chronological age, body adiposity, lifestyle factors, and cortisol levels were controlled for. Results showed no relationship between AR CAGn and biological age markers, directly or through interaction with T levels. Findings suggest that lifetime testosterone fluctuations, influenced by environmental and health factors, may obscure the link between AR CAGn and reproductive/somatic investment, ultimately affecting the aging rate.

**Supplementary Information:**

The online version contains supplementary material available at 10.1038/s41598-025-22090-3.

## Introduction

According to evolutionary biology theories, an individual’s health and aging depend on trade-offs between reproductive effort and soma maintenance^1^. Greater reproductive effort is expected to affect health and shorten lifespan^2,3^, contributing to substantial inter-individual variability in the aging process and age-related health outcomes^4^. Numerous studies confirm that higher reproductive effort is associated with poorer health and a shorter post-reproductive lifespan in both sexes^5–7^. However, other studies fail to establish this relationship or even suggest a positive correlation between reproductive effort and lifespan^8–10^. In men, these trade-offs between reproductive and somatic investment are generally thought to be regulated by androgens, reflecting the biological costs of elevated androgen levels, primarily testosterone (T) and 5-α dihydrotestosterone (DHT)^11^.

Androgens are pleiotropic hormones that exert a wide range of physiological effects on both the reproductive and non-reproductive systems of the male body across different life stages. Their influence begins in utero, contributing to dimorphic morphological, sexual, developmental, psychological, and behavioral characteristics. Androgens also play a significant role in sex-specific metabolic programming, resulting in different health risk profiles between men and women later in life^12^. In general, androgens enhance reproductive effort and intra-sexual competition by increasing attractiveness (through morphological masculinity), promoting mating behaviors, increasing libido, and fostering more aggressive competition for mates^13^. At the same time, androgens may negatively affect survival-related outcomes, potentially by reducing the capacity to mobilize immune responses, store energy in adipose tissue, maintain antioxidant defenses, and by heightening susceptibility to injuries^11,14–19^. However, the relationship between androgen levels, health, and aging is complex.

Experimental and animal studies suggest that high androgen levels incur biological costs that may contribute to faster aging[e.g., ^14,15^]. This notion is supported by studies showing extended lifespans in castrated men^20^ and evidence that T abuse among athletes can lead to severe adverse effects and premature death^21^. Conversely, low T levels have also been linked to increased all-cause mortality, although a meta-analysis of 12 community-based surveys revealed considerable inconsistency across studies^22^. Furthermore, while men generally show increased susceptibility to infections^23^, Nowak et al.^24^ did not find that androgen levels suppressed immune function in men assessed by comprehensive measures. Similarly, although males are more susceptible to oxidative stress (OS) and have lower antioxidant capacity than females^25^, low T levels may also exacerbate OS^26^, and T replacement therapy seems to decrease OS in aging men^27^. These contradictions extend to the role of androgens in cardiometabolic health. Testosterone has been suggested to contribute to higher rates of atherosclerosis and cardiac disease in men compared to women and may influence a range of risk factors that increase susceptibility to these diseases^28–30^. However, age-related T decline is also linked to adverse health outcomes in men, and replacement therapy has demonstrated beneficial effects on cardiometabolic parameters. Despite some side effects, its overall impact on mortality appears to be largely positive^18,31,32^. Nevertheless, it is worth noting that the threshold at which symptoms of decreasing T levels begin to manifest varies greatly among individuals, with many men remaining asymptomatic despite very low T levels^33^.

In this complex scenario, surprisingly little is known about the potential role of the androgen receptor (AR) sensitivity in the life-history trade-offs regulated by androgen levels. Androgens exert their effects by activating ARs, which are widely distributed across body tissues^34^. Exon 1 of the AR gene contains two polymorphic triplet repeats, CAG and GGN, which encode polyglutamine and polyglycine tracts, respectively^35^. The length of the CAG repeats ranges from 8 to 35, with an average of about 22^36^. Studies indicate that shorter CAG repeats are linked to increased *AR* protein expression (i.e., higher receptor density) as well as enhanced binding affinity and sensitivity of the androgen receptor to T and DHT. In contrast, longer CAG repeats reduce receptor sensitivity^37,38^. As a result, men with shorter CAG sequences may display stronger physiological responses to the same circulating androgen levels than men with longer repeats^39^. Thus, the CAG repeat number likely has an inverse relationship with AR sensitivity, potentially influencing individual differences in physical traits, cognitive abilities, and behaviors. This variation could also potentially predict inter-individual differences in the inclination toward greater mating effort and susceptibility to disorders related to androgen activity^40^.

The AR CAG repeat length has been linked to various fitness and health outcomes, including male reproductive function, muscle mass, cardiovascular health, prostate cancer risk, and bone density^41–44^, although findings remain inconsistent^45^. For example, men with a lower number of CAG repeats (< 22) as well as those with a higher number (> 23) showed a trend toward increased mortality, suggesting a U-shaped relationship between AR CAG repeat length and mortality^46^. Furthermore, shorter AR CAG repeats have been associated with an increased risk and earlier onset of prostate cancer^47^. However, other studies have not found this effect^48^. In addition, shorter AR CAGn has been associated with both protective cardiometabolic factors (e.g., low body fat and plasma insulin) and adverse parameters (including low HDL cholesterol, higher blood pressure, and increased visceral fat)^49–52^. Yet, several studies reported no relationship between AR CAG repeat length and these health outcomes^53^.

Despite some inconsistencies, the AR gene appears to play an important role in the development and maintenance of masculine traits^54^, male reproduction^55^, and health^46,49–52^. AR sensitivity may therefore be crucial for understanding testosterone-dependent life-history trade-offs and their implications for biological condition and aging. Higher T levels and shorter AR CAGn repeats may predispose individuals to increased reproductive investment, potentially contributing to faster aging. Yet relatively few studies have examined the association between AR CAG repeat length and markers of biological aging. For example, experimental studies have shown that AR overexpression enhances the expression of anti-aging klotho mRNA and protein, whereas T-induced klotho expression is attenuated in the presence of flutamide, an AR antagonist ^56^. This suggests that AR expression may be positively related to klotho levels; however, no research has investigated the relationship between AR sensitivity and klotho levels in men. Similarly, although androgens impact inflammatory processes due to their immunosuppressive activity^57,58^, no research has directly tested whether AR CAG repeat length is linked to inflammation, a key component of the aging process^59^. Interestingly, shorter CAG repeats have been reported to provide protection against severe COVID-19, which may point to a potential anti-inflammatory effect^60^.

This study aimed to investigate whether AR CAGn is associated with biological age markers in men. We hypothesized that shorter AR CAGn, indicating higher AR sensitivity, is associated with a higher propensity to prioritize reproductive effort over somatic investment, and thus to a more advanced biological age. In addition to AR CAGn repeats, we examined the independent effects of androgen levels (total and free testosterone) and their interaction with receptor sensitivity. We hypothesized that higher androgen levels, also indicative of greater reproductive investment, would also contribute to faster aging, although their effects may depend on AR sensitivity. Previous studies have suggested possible interactions between T levels and AR CAGn in T-dependent phenotypes, though findings remain inconsistent. For instance, while some studies report no direct association between CAGn and cardiometabolic risk factors, longer CAGn combined with low T levels has been linked to an increased risk of metabolic syndrome^61^. In contrast, other studies found no relationship between androgens, AR CAG repeat polymorphism, frailty syndrome, or the rate of phenotypic aging^62^. Based on previous findings, we predicted that the most rapid aging would occur in individuals with shorter CAG repeats and higher T levels, and the slowest in those with longer CAG repeats and lower T levels. We further suggest that, in some individuals, shorter AR CAGn may be offset by lower testosterone levels. Finally, given evidence suggesting non-linear AR CAGn activity depending on repeat length^55,63^, we also considered potential non-linear associations between AR CAGn and biological age markers.

The study was conducted in a sample of relatively young adult men (30–45 years), a reproductive-age group largely free from age-related conditions, yet still showing individual variability in aging, to capture individual differences in biological aging within a healthy population. Previous research demonstrates that aging trajectories can be detected as early as the 20s, with clear differences in aging rates and their implications for healthspan^64–66^. This highlights the importance of studying younger cohorts, as geroprotective interventions must be implemented by midlife to effectively prevent the onset of age-related diseases^65,67^.

Biological age was assessed based on a range of physiological markers, including klotho levels, inflammatory markers, oxidative stress, total antioxidant capacity, and DHEA/S levels. The klotho gene functions as an aging-suppressor gene^68^ and is widely used as a marker of biological age^69^. Aging is also associated with immune dysfunction, leading to a low-grade chronic inflammatory state, which serves as another marker of biological age^70^. Additionally, oxidative stress increases and accumulates with age, causing progressive cellular damage and functional decline; thus, products of oxidative damage are important physiological markers of biological age^71^. Dehydroepiandrosterone (DHEA) and its sulfated metabolite (DHEA-S) are key hormonal markers of biological age, as their concentrations typically decrease steadily with age, albeit with considerable inter-individual variability^72^.

In the analyses, we controlled for chronological age and other factors that may impact biological age, including body adiposity^73^, and lifestyle variables such as past smoking^74^, physical activity^75^, alcohol consumption patterns^76^, and socioeconomic status^77^. Given the significant role of cortisol in the pathophysiological mechanisms of aging, it was also included as a control variable^78^.

## Methods

This study was part of a project investigating factors related to men’s health, which included 209 men (M_age_ = 36.14, SD_age_ = 3.53 years) recruited from an urban population in Poland. All methods followed relevant guidelines and regulations and were reviewed and approved by the Commission of Bioethics at Wroclaw Medical University (nr 222/2019). All procedures were consistent with the guidelines included in the “Declaration of Helsinki—Ethical Principles for Medical Research Involving Human Subjects” formulated by the World Medical Association. All participants signed written informed consent for participation in the study, including consent for genetic analyses.

### Participants

Participants were recruited through announcements in local newspapers, the University’s social media channels, and local radio. Buccal swabs for genetic analyses were collected from 140 men (M_age_ = 35.35, SD_age_ = 3.59). Nine participants were excluded for the following reasons: (1) regular smoking (N = 4), (2) CRP > 10 mg/L, indicating ongoing inflammation (N = 1), (3) chronic diseases (N = 1), and (4) incomplete data (N = 3). Health status was evaluated based on inflammation markers, blood morphology, and self-reported health, and none of the participants exhibited symptoms of ongoing infection. The final analyses were conducted on 131 healthy men (M_age_ = 35.45, SD_age_ = 3.66), with ages ranging from 29.96 to 44.29 years.

### Procedures

A fasting blood sample was drawn between 7:30 a.m. and 9:00 a.m. for biochemical and hormone analyses, including markers of biological age. Participants were asked to refrain from physical activity, heavy meals, and alcohol for 24 h before the visit. Body adiposity was measured using bioimpedance analysis (SECA mBCA 515).

The participants completed a questionnaire collecting information on demographics, current and past health issues, and medication use. The questionnaire also included items on lifestyle factors that could influence biological age, such as smoking, alcohol consumption, physical activity, and socioeconomic status. Among the participants, 18 reported being former regular smokers (having quit at least one year before the visit), while 113 stated they had never smoked. Based on the reported frequency of alcohol consumption, participants were grouped as follows: (1) rarely—once per month or less (N = 33), (2) sometimes—2–4 times per month (N = 60), and (3) often—2–3 times per week (N = 38). Participants provided details about their physical activity, including the frequency, duration (in minutes) of weekly training sessions, and the type of sport practiced. Reported activities were of comparable intensity and included running, cycling, swimming, football, basketball, calisthenics, tennis, squash, CrossFit, and strength training. Participants were categorized as (1) physically active (N = 80), engaging in at least 60 min of regular physical activity per week, or (2) inactive (N = 51), reporting no regular sport activity. There were no professional athletes in the study sample.

Participants were asked to complete a subjective socioeconomic status (SES) measure^79^. They rated their agreement with the following statements on a scale from 1 (strongly disagree) to 7 (strongly agree): (a) “I don’t worry too much about paying my bills,” (b) “I have enough money to buy the things I want,” and (c) “I don’t expect to worry about money much in the future”. An average of the three ratings was used to calculate an overall subjective SES score.

### AR CAG repeats analysis

Genetic material was isolated from participants’ buccal swabs using proteinase K digestion followed by phenol–chloroform extraction, as described by Sambrook et al.^80^. DNA concentration was measured using a UV/VIS spectrophotometer Colibri Microvolume Spectrometer (Titertek-Berthold, Germany).

To investigate the number of CAG repeats in the AR gene promoter, we have constructed a set of primers flanking the NM_000044.6(AR): c.171GCA[10_36] (rs3032358) region. Primer specificity for the androgen receptor repeat instability region was verified using the Primer-BLAST tool from NCBI (https://www.ncbi.nlm.nih.gov/tools/primer-blast/). Oligonucleotides were extended with standard sequences of bacteriophages M13 and T7, facilitating sequencing and stabilizing primers during PCR.

PCR amplifications were performed using StartWarm HS-PCR Mix (2x) (A&A Biotechnology, Poland), which includes Hot-Start Taq DNA polymerase, in a T100 Thermal Cycler (Bio-Rad). The final volume of 50 μl of each reaction contained 0.25 μM of each primer (forward: 5′-GTAAAACGACGGCCAGT-AGCTTTCCAGAATCTGTTCCAG-3′; reverse: 5′-TAATACGACTCACTATAGG-AGAACCATCCTCACCCTGCT-3′) (Sigma-Aldrich, UK) and 80–100 ng of the genomic DNA. Amplification conditions were as follows: initial denaturation at 94 °C for 10 min; 40 cycles of denaturation at 94 °C for 30 s, annealing at 58 °C for 30 s, and extension at 72 °C for 30 s; with a final extension at 72 °C for 5 min. PCR products (262 ± 39 bp) were sequenced by Sanger sequencing using an Applied Biosystems 3130xl Genetic Analyzer.

### Biological age markers measurement

The blood samples were centrifuged, and serum was collected and stored at -80°C until analysis with enzyme-linked immunosorbent assays (ELISA). The procedures followed the manufacturers’ protocols. Serum samples, calibrators (standards supplied with a kit), and controls (if provided with the kit) were tested in duplicate. Absorbance was measured with a microplate reader (ASUS UVR340) at the wavelength specified in each kit’s manual. The standard curve was created by plotting the mean absorbance values for each standard (Y-axis) against its concentration (X-axis). The results were calculated using this curve to determine the concentration corresponding to each sample’s absorbance value. The average concentration of the two measurements was used in the analysis.

Serum soluble circulating Klotho (s-Klotho) levels were measured using a commercial kit (IBL® Code no 27998) and expressed in pg/ml. The inter-assay and intra-assay coefficients of variation were less than 11.4% and 3.5%, respectively, with an assay sensitivity of 6.15 pg/ml. Serum DHEA and DHEA-S levels were assayed using a DEMEDITEC competitive ELISA kit. DHEA (cat no DEH3344) level was measured with inter- and intra-CV less than 6.9%. DHEA-S (cat no DEH3366) level was measured with inter- and intra-CV less than 12.2% and less than 6.8%, respectively. Serum hs-CRP level was measured using a DEMEDITEC ELISA kit (cat no 740011) with inter- and intra-CV less than 6.3% and 6.9%, respectively. Serum hsIL-6 level was measured using an R&D Systems ELISA kit (cat no HS600C) with inter- and intra-CV less than 10.8% and less than 4.7%, respectively.

Serum 8-epi-PGF2α level was assayed with an Elabscience competitive-ELISA kit (cat. No E-EL-0041) with intra- and inter-CV less than 7%. Protein carbonyls were assayed with a MyBioSource ELISA kit (cat. No MBS3802635). DNA/RNA oxidative damage was assayed with a Cayman ELISA kit (cat. No 589320) with inter- and intra-CV less than 12%. The kit allows the measurement of DNA/RNA oxidative damage, assesses 8-OH-dG from DNA, 8-OHG from RNA, and 8-hydroxyguanine from either DNA or RNA. As the three markers reflect the same underlying process, albeit capture damage to different molecular targets, an aggregate measure combining these indicators was calculated. Such a composite measure of oxidative stress is expected to provide a more accurate assessment of overall oxidative status than a single biomarker^81^.

Serum total antioxidant capacity (TAC) was assayed with a Cayman ELISA kit (cat. No 709001), with an inter-assay CV of 3% and an intra-assay CV of 3.4%. The kit enables the assessment of both aqueous- and lipid-soluble antioxidants; thus, the combined antioxidant capacities of all its constituents, including vitamins, proteins, lipids, glutathione, and uric acid, were evaluated.

### Cortisol, estradiol, and testosterone measurement

Serum cortisol level was measured using a DEMEDITEC ELISA kit (cat no DEH3388) with inter- and intra-CV less than 9.2% and less than 7.2%, respectively.

Total (tT) and free testosterone (fT) levels were measured by a certified analytical laboratory (DIAGNOSTYKA®) using a Cobas analyzer and reported in ng/ml and pg/ml, respectively.

### Statistical analysis

Normality was assessed using the Kolmogorov–Smirnov test, as well as kurtosis, skewness, and visual inspection of plots. The distributions of age, DHEAS-S, 8-epi-PGF2α, cortisol, biological age index, and body adiposity were considered normal, as the z-scores for kurtosis and skewness were below 3.29^82^. Total testosterone, free testosterone, Klotho, DHEA, hsIL-6, and RNA/DNA levels were log-transformed due to positive Kolmogorov–Smirnov test results and high levels of kurtosis and skewness. The distributions of AR CAGn, hsCRP, PCs, oxidative stress index, TAC, E2, and SES deviated from normality; therefore, non-parametric tests were used in the analyses involving these variables. The dataset is available in the supplementary material (Table S1).

An aggregate measure of oxidative stress was calculated by Z-scoring each OS biomarker (8-epi-PGF2α, protein carbonyls, DNA/RNA oxidative damage, each reflecting different outcomes of oxidative stress) and averaging the three Z-scores for each individual. Similarly, an aggregate measure of biological age was calculated by Z-scoring each biomarker (s-Klotho, hs-CRP, hsIL-6, DHEA, 8-isoepiprostaglandine, RNA/DNA, PCs, TAC levels) and averaging the seven Z-scores for each individual. The Z scores of s-Klotho, DHEA, and TAC were multiplied by − 1 to account for their inverse relationship with biological age. Higher values of this composite measure indicated an older biological age.

First, the relationship between AR CAGn and biological age marker levels was verified with Spearman correlation analysis. To examine potential non-linear effects, participants were divided into three groups based on CAG repeat number: 1) short repeats, CAG < 21 (N = 47); 2) average repeats, CAG = 21–22 (N = 35); 3) long repeats, CAG > 22 (N = 49). ANOVA was used to compare the values of biological age markers across these three groups. Finally, to directly test for curvilinear associations, regression analyses were conducted, including a squared AR CAGn term. AR CAGn values were mean-centered before creating the quadratic term to reduce multicollinearity between the linear and squared components. Together, these approaches allowed us to comprehensively assess potential quadratic effects of AR CAGn on biological age markers.

The General Linear Model was used to examine associations between AR CAG repeat length and biological age markers, while controlling for other factors known to influence biological aging, such as chronological age, body adiposity, alcohol use, cortisol level, physical activity, and SES. Control variables were included in each regression model only if they showed a significant correlation with the specific biological age marker, resulting in slightly different covariate sets across models. The t-test was used to determine whether participants who smoked in the past (N = 18) differed from those who had never smoked (N = 113) in terms of markers of biological age. A t-test was also conducted to examine whether physically active participants (N = 80) differed from inactive participants (N = 51) on the same markers. ANOVA was used to assess differences in biological age markers according to alcohol consumption frequency: (1) rarely (N = 33), (2) sometimes (N = 60), and (3) often (N = 38), with the Tukey test applied as a post-hoc analysis. Pearson and Spearman correlations were used to verify whether AR CAGn and biological age markers were associated with continuous controlled variables (chronological age, body adiposity, cortisol, and SES). Thus, the predictors for each analysis were selected based on the t-test and Pearson correlation analysis results for the relationship between biological age markers and potential confounders.

We also tested whether testosterone moderated the association between AR CAGn and biological age markers. Potential moderation was tested within the General Linear Model framework by including the interaction term between testosterone and AR CAG repeat length (T × CAGn).

All analyses were conducted in JAMOVI, and results were considered statistically significant at *p* < 0.05.

## Results

### Descriptive statistics

Descriptive statistics are presented in Table 1. The distribution of AR CAG repeat numbers was consistent with previous reports from the Polish population^83^, with 21 repeats being the most common (16.8%).

**Table 1:** Descriptive statistics of the studied variables (N = 131).

	M	SD	Min	Max
Age [years]	35.45	3.66	29.96	44.29
AR CAG_n_	21.34	3.90	7.00	32.00
Cortisol [ng/ml]	317.82	62.11	149.00	516.93
Total testosterone [ng/dl]	504.53	173.46	146.90	1157.00
Free testosterone [pg/ml]	15.17	5.42	4.88	35.55
Adiposity [%]	22.19	6.58	6.07	42.33
Klotho [pg/l]	1199.83	501.03	306.70	2686.50
DHEA [ng/ml]	9.13	5.83	1.15	41.78
DHEA-S [ug/ml]	4.07	1.09	0.94	6.28
hsIl-6 [pg/ml]	2.65	2.71	0.29	12.94
hs-CRP [pg/ml]	1.07	1.05	0.01	4.51
8-epi-PGF2α [pg/ml]	206.35	71.78	103.71	414.42
RNA/DNA [pg/ml]	18,540.82	7006.47	6209.10	37,793.63
PCs [ng/ml]	35.55	23.32	2.85	244.13
TAC [um]	2.05	0.32	1.10	2.71
Oxidative stress index	0.00	0.58	-0.83	3.79
Biological age index	0.002	0.36	-1.04	1.27

Men who smoked in the past (N = 18) and men who never smoked (N = 113) did not differ in terms of biological age measures (in each case, *p* > 0.16); therefore, smoking status was not included as a control variable in further analyses. Physically active men (N = 80) had higher levels of DHEA (M_DHEA_ = 9.87 ± 6.37) and lower levels of hs-CRP (M_hsCRP_ = 0.93 ± 0.94) compared to men who were not active (M_DHEA_ = 7.97 ± 7.03; M_hsCRP_ = 1.30 ± 1.17; N = 51) (DHEA: t(129) = − 2.00, *p* = 0.049; hs-CRP: U = 1615, *p* = 0.045). The two groups did not differ in terms of other measures of biological age (in each case, *p* > 0.14). Consequently, physical activity was controlled only in models that included DHEA or hsCRP as dependent variables. Alcohol consumption frequency was negatively related to s-klotho level (F(2,128) = 8.18, *p* < 0.001) and positively to RNA/DNA oxidative damage level (F(2128) = 6.01, *p* = 0.003). No associations were found with other biological age markers (in each case, *p* > 0.13). Therefore, alcohol consumption was controlled only in analyses involving s-klotho or RNA/DNA oxidative damage as dependent variables. Chronological age was negatively related to DHEA/S levels, but not to other measures of biological age, including total and free testosterone, or AR CAGn. Therefore, age was controlled only in the analyses that included DHEA/S as the dependent variable. Body adiposity was negatively related to DHEA and positively to hs-CRP, oxidative stress markers, and biological age index. It was also negatively related to total and free testosterone. Consequently, body adiposity was controlled in all analyses that included these variables. Cortisol showed a strong positive correlation with DHEA/S and a negative correlation with the biological age index. This pattern likely reflects their shared regulation by the adrenal cortex, as both hormones are secreted in response to ACTH^84^, rather than a direct effect of cortisol on biological age markers. No associations were also found between cortisol and total/free testosterone or AR CAGn. Therefore, cortisol was not included as a control variable in subsequent analyses. SES was unrelated to biological age markers, testosterone (total or free), or AR CAGn, but showed a marginal negative association with the oxidative stress index (*p* = 0.051). Therefore, SES was controlled only in the analyses involving the oxidative stress index (Table 2).

**Table 2:** Correlation results for the relationship between biological age markers and controlled variables (N = 131).

	Age [years]		Adiposity [%]		Cortisol [ng/ml]		SES	
	r/ρ	*P*	r/ρ	*P*	r/ρ	*P*	ρ	*p*
Klotho log [pg/l]	0.12	0.16	0.04	0.70	0.09	0.31	0.03	0.75
DHEA log [ng/ml]	**-0.29**	** < 0.001**	**-0.21**	**0.02**	**0.62**	** < 0.001**	0.03	0.75
DHEA-S [ug/ml]	**-0.21**	**0.02**	-0.02	0.80	**0.22**	**0.01**	0.04	0.71
hsIl-6 log [pg/ml]	0.06	0.47	0.09	0.29	-0.01	0.91	-0.07	0.45
hs-CRP [pg/ml]^1^	0.07	0.46	**0.47**	** < 0.001**	**-0.30**	** < 0.001**	-0.03	0.75
8-epi-PGF2α [pg/ml]	0.13	0.16	-0.10	0.24	-0.02	0.82	-0.17	0.08
RNA/DNA log [pg/ml]	-0.03	0.76	**0.22**	**0.01**	-0.06	0.47	-0.01	0.95
PCs [ng/ml]^1^	-0.07	0.45	-0.02	0.85	0.02	0.84	-0.10	0.31
TAC [um]^1^	0.03	0.75	-0.01	0.88	0.09	0.33	0.05	0.62
OS index^1^	0.11	0.23	0.04	0.64	0.11	0.23	-0.19	0.05
Biological age index	0.12	0.16	**0.24**	**0.007**	**-0.39**	** < 0.001**	-0.15	0.11
Total T log [ng/dl]	-0.14	0.11	**-0.43**	** < 0.001**	0.10	0.28	0.09	0.28
Free T log [pg/ml]	-0.15	0.09	**-0.31**	** < 0.001**	0.12	0.19	0.07	0.44
AR CAGn	-0.11	0.21	0.03	0.77	0.11	0.23	-0.05	0.56

^1^ Spearman correlation.

Significant values are bold.

### The relationship between AR CAGn and markers of biological age

We found no correlation between AR CAGn and biological age measures. Similarly, biological age markers were unrelated to total and free testosterone levels (Table 3).

**Table 3:** Correlation results for the relationship between AR CAGn and biological age markers (N = 131).

	AR CAGn		LOG testosterone [ng/dl]		LOG free testosterone [pg/ml]	
	ρ	*p*	r/ρ	*p*	r/ρ	*p*
Klotho log [pg/l]	0.13	0.15	0.02	0.85	0.005	0.95
DHEA log [ng/ml]	− 0.03	0.76	0.13	0.15	− 0.05	0.60
DHEA-S [ug/ml]	− 0.005	0.95	0.03	0.73	− 0.08	0.35
hsIl-6 log [pg/ml]	0.004	0.97	0.002	0.99	0.07	0.42
hs-CRP [pg/ml]	0.004	0.96	− 0.08^1^	0.40	− 0.01^1^	0.90
8-epi-PGF2α [pg/ml]	− 0.04	0.63	0.10	0.25	0.11	0.20
RNA/DNA log [pg/ml]	− 0.001	0.99	− 0.12	0.17	− 0.11	0.22
PCs [ng/ml]	0.06	0.48	0.02^1^	0.85	− 0.07^1^	0.43
TAC [um]	− 0.06	0.53	− 0.09^1^	0.32	− 0.09^1^	0.32
OS index	0.13	0.15	− 0.01	0.94	0.003	0.97
Biological age index	− 0.03	0.77	0.03	0.76	0.04	0.68

^1^ Spearman correlation.

The results of ANOVA analyses showed no difference in terms of biological age markers between men with short (N = 47), average (N = 35), and long (N = 49) AR CAG repeats (in each case, *p* > 0.12). Also, the quadratic effect of CAG repeats length (CAGn^2^) reached no statistical significance (all *p* > 0.12), indicating no evidence for U-shaped or inverted U-shaped associations with biological age.

### The relationship between AR CAGn x testosterone interaction and markers of biological age

We found no simple moderation of AR CAGn for the relationship between biological age markers and total or free testosterone level (*p* > 0.05). We also found no significance for the interaction between total or free testosterone and AR CAGn in the relationship between AR CAGn or total or free testosterone level and biological age markers when controlled for body adiposity and cortisol level. Table 4 presents the results of the regression analysis with the biological age index as the dependent variable. Results for the other biological age markers are provided in the Supplementary Material (Tables S1-S10). Introducing alcohol use, smoking in the past, physical activity, or SES in the model did not improve model statistics.

**Table 4:** Regression analysis for the relationship between biological age index and AR CAGn, testosterone (Model 1) or free testosterone (Model 2), and the interaction between the two, controlled for body adiposity (N = 131).

	β	t (126)	*p*
Model 1	F(4,126) = 2.83, *adj. R*^*2*^ = 0.05, *p* = 0.03		
AR CAGn	− 0.03	− 0.39	0.70
T [ng/dl]	0.17	− 1.26	0.17
AR CAGn x T	− 0.10	− 1.26	0.21
Body adiposity [%]	**0.30**	**3.17**	**0.002**
Model 2	F(4,125) = 2.45, *adj. R*^*2*^ = 0.04, *p* = 0.05		
AR CAGn	− 0.03	− 0.28	0.78
fT [ng/dl]	0.14	1.50	0.14
AR CAGn x fT	− 0.07	− 0.73	0.47
Body adiposity [%]	**0.27**	**2.95**	**0.004**

Significant values are bold.

## Discussion

We found no evidence that the number of CAG repeats in exon 1 of the androgen receptor is related to men’s biological age, either directly or through interaction with baseline testosterone concentrations in men aged 30 to 45. Biological age markers were also unrelated to testosterone levels. These results remained consistent after controlling for body adiposity, socioeconomic status (SES), physical activity, alcohol use, and smoking history. Furthermore, although in vitro studies suggest that the relationship between AR CAG repeat length and T-dependent phenotypic effects may follow a U-shaped curve^63^, we found no evidence of non-linear effects in our study. Other research also shows that these non-linear effects may be weak. For instance, Tut et al.^55^ showed that while the longest repeat (31 CAG) exhibited decreased activity compared to the shortest one (15 CAG), neither shorter nor longer repeats differed significantly from the intermediate length (20 CAG) in their ability to activate the reporter gene.

This study is the first to specifically examine the relationship between AR CAG repeat length and biological age in men, making direct comparisons with prior research difficult. Nevertheless, our findings align with studies reporting no association between CAG repeat length and testosterone-dependent somatic traits that reflect long-term androgen exposure. For instance, AR CAG repeat length has been shown to be unrelated to the 2D:4D digit ratio^85,86^ and the facial width-to-height ratio (fWHR)^87^. On the other hand, other studies have found positive correlations between CAG repeat length and traits like sexual jealousy^88^, and negative correlations with dominance^13^, aggression^89^, muscle mass^90^, and upper body strength^13^. Evidence on AR CAG repeats and cardiometabolic risk, an important component of the aging phenotype, has also been inconsistent. While two large cross-sectional studies reported no associations with heart disease, HDL, LDL, or waist-to-hip ratio^53,61^, and other studies found no effect on lipid levels^91,92^, some research suggests otherwise. AR CAG repeat length has been linked to more severe coronary artery disease^92^ and to cardiovascular risk factors such as elevated LDL^93^ and reduced HDL^52^. Moreover, an inverse relationship between repeat length and insulin resistance (measured by HOMA-IR) has been observed ^94^. Previous research has also reported a positive, rather than negative, correlation between testosterone and the expression of the klotho gene^56^ and circulating s-klotho levels^86,95,96^. Taken together, these mixed findings underscore the complexity of how AR CAG repeat length interacts with biological aging and health outcomes.

The absence of a negative effect of androgen receptor sensitivity and androgen level on biological aging rate, as observed in our study, may be explained by the possibility that the costs of testosterone are mitigated by the ability to reduce baseline testosterone level as a function of AR sensitivity and of optimal developmental trade-offs^41,45,97,98^. We hypothesized that AR CAG repeat length may mediate trade-offs between somatic maintenance and reproductive investment, where shorter CAG repeats are linked to greater reproductive effort and investment. This would arise from greater androgen exposure and higher T costs, potentially contributing to faster aging. However, these trade-offs may be dynamically regulated through adjustments in circulating androgen levels. In our study, the interaction between AR CAG repeat length and baseline testosterone concentrations was also tested, but no significant effect was observed. This suggests that even individuals who simultaneously display higher testosterone levels and greater AR sensitivity do not exhibit accelerated biological aging. Nevertheless, since testosterone was measured only once, we cannot rule out the possibility that such interactions might emerge at other stages of life or under specific circumstances, such as during physical competition when testosterone levels temporarily rise. Testosterone levels fluctuate across contexts, tending to rise when reproductive opportunities are high (e.g., when single or in the presence of potential mates) and decline during periods of food scarcity or immune challenge^11,13^. Impairments in AR functionality can also be compensated through activation of the hypothalamic–pituitary–testis axis, resulting in elevated androgen production and/or regulation of AR expression. For instance, Nenonen et al.^63^ found a lower amount of AR mRNA and protein in the CAG22 variant than in those in the lower or upper normal range, indicating that at least within this CAG range, even in an experimental setting, differences in function can be compensated for by higher AR expression. Buchanan et al.^36^ also proposed that normal AR function is sustained within a critical, but narrow, range of CAG repeat lengths, which may help explain the conflicting results in studies examining AR CAG repeats and diseases like male infertility or prostate cancer.

The notion that high androgen levels entail biological costs is central to evolutionary theories on the adaptive significance of testosterone-dependent traits. Such traits are believed to indicate an individual’s biological condition, and the reliability of information conveyed by these traits is maintained by a cost involved in their development and maintenance, which can be afforded only by individuals in good biological condition^99^. The dynamic interplay between receptor sensitivity and androgen levels across ontogenesis may help explain the results of studies showing no biological cost associated with developing testosterone-dependent morphological traits[e.g., ^100–103^]. It might also explain the inconsistent findings regarding the relationship between AR CAG repeat length and testosterone-dependent characteristics.

Furthermore, although the androgen receptor (AR) is the only gene that has been shown so far to be linked to androgen insensitivity syndrome (AIS), the androgen signaling pathway is complex, with over 400 different AR mutations leading to AIS^104^. Other genetic variants, such as GGC repeat length in exon 1 of the AR gene^105^ or a single nucleotide polymorphism (SNP) involving a G-to-A transition at position 211 in exon 1 (rs6152)^106^, may also influence the aging profile. These polymorphisms appear to function independently, affecting distinct aspects of AR sensitivity and function, with no evidence suggesting they are correlated in terms of length or effects. In vitro studies indicate that each additional CAG repeat reduces testosterone effectiveness by approximately 2%, but the studies on the relationship between AR CAG repeat length and testosterone-dependent traits suggest that in vivo effects are more complex^107^. It is also important to recognize that androgens act through both genomic (direct) and non-genomic (indirect) mechanisms. While testosterone primarily signals via AR, some effects occur independently, such as through conversion to estrogens or rapid, non-transcriptional actions. Additionally, certain T metabolites may act independently of AR, interacting with different receptors or cellular pathways^108^. These factors might influence the significance of AR sensitivity for testosterone-dependent traits, further complicating the understanding of their relationship.

Interpreting these results requires an understanding of our study limitations. First, biological age markers were measured only once. Despite a rigorous study protocol, we cannot rule out the possibility of intra-individual variability in the markers. Future studies should include repeated measurements of biological age markers and incorporate additional markers of biological age. Second, the cross-sectional design limits our ability to exclude the influence of various factors that may affect the relationship throughout ontogeny. It’s important to note that lifestyle factors (such as obesity, physical inactivity, anabolic steroid use, stress, etc.) can influence hormonal levels, potentially masking the genetic effects of the AR CAG polymorphism in men^109^. However, it’s worth noting that, first, we found body adiposity to be positively related to biological age markers, suggesting adequate variability, and second, previous research has demonstrated biological aging differences in both young and middle-aged cohorts^110–112^.

As human lifespan has markedly increased in recent decades, understanding the factors underlying variability in biological age has become increasingly relevant^113^. In this study, we examined whether life-history trade-offs, largely regulated by androgen levels, contribute to individual differences in biological aging, recognizing that despite recent advances, our understanding of the mechanisms driving these trade-offs and individual differences in aging and healthspan remains incomplete. Despite theoretical expectations, we found no association between AR CAG repeat length, testosterone levels, their interaction, and biological age markers in men aged 30 to 45. One possible explanation is that fluctuations in testosterone across life, shaped by environmental and health factors, obscure stable relationships between AR sensitivity and reproductive or somatic investment, and thus biological aging. Interestingly, AR CAG repeats have accumulated almost exponentially throughout mammalian evolution^114^ and continue to do so in humans, even though longer repeats are linked to certain pathologies. This suggests that shorter and longer AR CAG repeats may provide distinct advantages, potentially balanced by hormonal adjustments that compensate for AR sensitivity. This may complicate the impact of AR sensitivity on various body functions and processes, including the rate of biological aging.

## Supplementary Information

Below is the link to the electronic supplementary material.

Supplementary Material 1

Supplementary Material 2

## Data Availability

The datasets analyzed in this study are available in the supplementary material.
