# Supplementary material for: Androgen receptor gene polymorphism and biological age markers in men
Source: Sci Rep. 2025 Oct 31;15:38226. doi: 10.1038/s41598-025-22090-3 (PMC12578872; doi:10.1038/s41598-025-22090-3)
Supplement: Supplementary file 2 — Supplementary Material 2 [file 41598_2025_22090_MOESM2_ESM.docx]

**Supplementary material**

Table S1. Regression analysis for the relationship between klotho level and AR CAGn, testosterone (Model 1) or free testosterone (Model 2), and the interaction between the two, controlled for body adiposity and alcohol consumption (N = 131). Bolded values are statistically significant.

|  | β | t | *p* |
| --- | --- | --- | --- |
| Model 1 | F(5,125) = 4.10, *adj. R^2^* = 0.11, *p* = 0.002 | | |
| AR CAGn | -0.02 | -0.19 | 0.85 |
| LOG T [ng/dl] | -0.001 | -0.01 | 0.99 |
| AR CAGn x T | 0.15 | 1.86 | 0.07 |
| **Alcohol consumption*** | **-0.35** | **-4.16** | **<0.001** |
| Body adiposity [%] | 0.10 | 1.06 | 0.29 |
| Model 2 | F(5,124) = 3.70, *adj. R^2^* = 0.10, *p* = 0.004 | | |
| AR CAGn | -0.01 | -0.14 | 0.89 |
| LOG fT [ng/dl] | 0.04 | 0.43 | 0.67 |
| AR CAGn x fT | 0.13 | 1.42 | 0.16 |
| **Alcohol consumption*** | **-0.35** | **-4.06** | **<0.001** |
| Body adiposity [%] | 0.11 | 1.26 | 0.21 |

* Coded as: once per month or less = 0; 2–4 times per month = 1; 2–3 times per week = 2

Table S2. Regression analysis for the relationship between DHEA level and AR CAGn, testosterone (Model 1) or free testosterone (Model 2), and the interaction between the two, controlled for body adiposity (N = 131).

|  | β | t | *p* |
| --- | --- | --- | --- |
| Model 1 | F(4,126) = 1.75, *adj. R^2^* = 0.03, *p* = 0.13 | | |
| AR CAGn | 0.04 | 0.42 | 0.68 |
| LOG T [ng/dl] | 0.01 | 0.09 | 0.93 |
| AR CAGn x T | 0.05 | 0.59 | 0.56 |
| Physical activity* | 0.12 | 1.39 | 0.17 |
| Body adiposity | -0.18 | -1.83 | 0.07 |
| Model 2 | F(4,125) = 1.79, *adj. R^2^* = 0.03, *p* = 0.12 | | |
| AR CAGn | 0.01 | 0.13 | 0.90 |
| LOG fT [ng/dl] | 0.10 | 1.06 | 0.29 |
| AR CAGn x fT | -0.02 | -0.15 | 0.88 |
| Physical activity* | 0.12 | 1.28 | 0.20 |
| Body adiposity | -0.15 | -1.66 | 0.10 |

* Coded as: Physically inactive = 0; Physically active = 1

Table S3. Regression analysis for the relationship between DHEA-S level and AR CAGn, testosterone (Model 1) or free testosterone (Model 2), and the interaction between the two, controlled for body adiposity (N = 131). Bolded values are statistically significant.

|  | β | t | *p* |
| --- | --- | --- | --- |
| Model 1 | F(5,125) = 1.30, *adj. R^2^* = 0.01, *p* = 0.27 | | |
| AR CAGn | -0.01 | -0.09 | 0.93 |
| LOG T [ng/dl] | -0.03 | -0.27 | 0.79 |
| AR CAGn x T | 0.07 | 0.79 | 0.43 |
| **Chronological age** | **-0.22** | **-2.48** | **0.01** |
| Body adiposity [%] | -0.02 | -0.17 | 0.87 |
| Model 2 | F(5,124) = 2.15, *adj. R^2^* = 0.04, *p* = 0.06 | | |
| AR CAGn | -0.04 | -0.45 | 0.66 |
| LOG fT [ng/dl] | 0.18 | 1.91 | 0.06 |
| AR CAGn x fT | 0.09 | 0.93 | 0.35 |
| **Chronological age** | **-0.20** | **-2.22** | **0.03** |
| Body adiposity [%] | 0.06 | 0.63 | 0.53 |

Table S4. Regression analysis for the relationship between hsIl-6 level and AR CAGn, testosterone (Model 1) or free testosterone (Model 2), and the interaction between the two, controlled for body adiposity (N = 131).

|  | β | t | *p* |
| --- | --- | --- | --- |
| Model 1 | F(4,126) = 0.83, *adj. R^2^* = -0.005, *p* = 0.51 | | |
| AR CAGn | -0.01 | -0.06 | 0.96 |
| LOG T [ng/dl] | 0.06 | 0.57 | 0.57 |
| AR CAGn x T | -0.12 | -1.46 | 0.15 |
| Body adiposity [%] | 0.11 | 1.12 | 0.26 |
| Model 2 | F(4,125) = 1.07, *adj. R^2^* = 0.002, *p* = 0.37 | | |
| AR CAGn | -0.03 | -0.29 | 0.77 |
| LOG fT [ng/dl] | 0.12 | 1.22 | 0.23 |
| AR CAGn x fT | -0.14 | -1.43 | 0.15 |
| Body adiposity [%] | 0.12 | 1.24 | 0.12 |

Table S5. Regression analysis for the relationship between hsCRP level and AR CAGn, testosterone (Model 1) or free testosterone (Model 2), and the interaction between the two, controlled for body adiposity (N = 131). Bolded values are statistically significant.

|  | β | t | *p* |
| --- | --- | --- | --- |
| Model 1 | F(5,126) = 7.82, *adj. R^2^* = 0.21, *p* < 0.001 | | |
| AR CAGn | -0.10 | -1.25 | 0.22 |
| **LOG T [ng/dl]** | **0.19** | **2.05** | **0.04** |
| AR CAGn x T | -0.05 | -0.62 | 0.54 |
| Physical activity* | -0.08 | -1.02 | 0.31 |
| **Body adiposity** | **0.51** | **5.79** | **<0.001** |
| Model 2 | F(5,125) = 7.71, *adj. R^2^* = 0.21, *p* < 0.001 | | |
| AR CAGn | -0.10 | -1.24 | 0.22 |
| LOG fT [ng/dl] | 0.17 | 2.00 | 0.05 |
| AR CAGn x fT | -0.05 | -0.52 | 0.61 |
| Physical activity* | -0.09 | -1.09 | 0.28 |
| **Body adiposity** | **0.48** | **5.71** | **<0.001** |

* Coded as: Physically inactive = 0; Physically active = 1

Table S6. Regression analysis for the relationship between Oxidative stress index level and AR CAGn, testosterone (Model 1) or free testosterone (Model 2), and the interaction between the two, controlled for body adiposity (N = 131).

|  | β | t | *p* |
| --- | --- | --- | --- |
| Model 1 | F(5,125) = 0.52, *adj. R^2^* = -0.02, *p* = 0.76 | | |
| AR CAGn | -0.002 | -0.02 | 0.98 |
| LOG T [ng/dl] | 0.03 | 0.32 | 0.75 |
| AR CAGn x T | 0.03 | 0.35 | 0.73 |
| Body adiposity [%] | 0.05 | 0.49 | 0.62 |
| LOG SES | -0.12 | -1.38 | 0.17 |
| Model 2 | F(5,124) = 0.48, *adj. R^2^* = -0.02, *p* = 0.79 | | |
| AR CAGn | 0.004 | 0.05 | 0.96 |
| LOG fT [ng/dl] | 0.03 | 0.32 | 0.75 |
| AR CAGn x fT | 0.03 | 0.26 | 0.80 |
| Body adiposity [%] | 0.04 | 0.44 | 0.66 |
| LOG SES | -0.13 | -1.40 | 0.16 |

Table S7. Regression analysis for the relationship between RNA/DNA oxidative damage level and AR CAGn, testosterone (Model 1) or free testosterone (Model 2), and the interaction between the two, controlled for body adiposity (N = 131). Bolded values are statistically significant.

|  | β | t | *p* |
| --- | --- | --- | --- |
| Model 1 | F(5,125) = 2.74, *adj. R^2^* = 0.06, *p* = 0.02 | | |
| AR CAGn | 0.05 | 0.62 | 0.54 |
| LOG T [ng/dl] | -0.03 | -0.30 | 0.76 |
| AR CAGn x T | 0.06 | 0.76 | 0.45 |
| **Alcohol consumption*** | **0.24** | **2.77** | **0.01** |
| Body adiposity [%] | 0.15 | 1.55 | 0.12 |
| Model 2 | F(5,124) = 2.79, *adj. R^2^* = 0.07, *p* = 0.02 | | |
| AR CAGn | 0.06 | 0.70 | 0.48 |
| LOG fT [ng/dl] | -0.07 | -0.70 | 0.48 |
| AR CAGn x fT | 0.05 | 0.57 | 0.57 |
| **Alcohol consumption*** | **0.25** | **2.87** | **0.01** |
| Body adiposity [%] | 0.14 | 1.55 | 0.12 |

* Coded as: once per month or less = 0; 2–4 times per month = 1; 2–3 times per week = 2

Table S8. Regression analysis for the relationship between 8-epi-PGF2α level and AR CAGn, testosterone (Model 1) or free testosterone (Model 2), and the interaction between the two, controlled for body adiposity (N = 131).

|  | β | t | *p* |
| --- | --- | --- | --- |
| Model 1 | F(5,125) = 0.73, *adj. R^2^* = 0.01, *p* = 0.60 | | |
| AR CAGn | -0.09 | -0.95 | 0.34 |
| LOG T [ng/dl] | 0.09 | 0.90 | 0.37 |
| AR CAGn x T | 0.03 | 0.31 | 0.76 |
| Alcohol consumption* | -0.08 | -0.86 | 0.39 |
| Body adiposity [%] | -0.05 | -0.51 | 0.61 |
| Model 2 | F(5,124) = 0.91, *adj. R^2^* = -0.003, *p* = 0.47 | | |
| AR CAGn | -0.08 | -0.86 | 0.39 |
| LOG fT [ng/dl] | 0.11 | 1.21 | 0.23 |
| AR CAGn x fT | 0.04 | 0.39 | 0.70 |
| Alcohol consumption* | -0.10 | -1.08 | 0.28 |
| Body adiposity [%] | -0.06 | -0.59 | 0.56 |

* Coded as: once per month or less = 0; 2–4 times per month = 1; 2–3 times per week = 2

Table S9. Regression analysis for the relationship between PCs levels and AR CAGn, testosterone (Model 1) or free testosterone (Model 2), and the interaction between the two, controlled for body adiposity (N = 131).

|  | β | t | *p* |
| --- | --- | --- | --- |
| Model 1 | F(5,125) = 0.12, *adj. R^2^* = -0.04, *p* = 0.99 | | |
| AR CAGn | 0.06 | 0.68 | 0.50 |
| LOG T [ng/dl] | -0.03 | -0.33 | 0.74 |
| AR CAGn x T | -0.01 | -0.16 | 0.87 |
| Alcohol consumption* | 0.01 | 0.07 | 0.94 |
| Body adiposity [%] | -0.02 | -0.22 | 0.82 |
| Model 2 | F(4,124) = 0.15, *adj. R^2^* = -0.03, *p* = 0.98 | | |
| AR CAGn | 0.06 | 0.60 | 0.55 |
| LOG fT [ng/dl] | -0.03 | -0.31 | 0.76 |
| AR CAGn x fT | -0.05 | -0.46 | 0.65 |
| Alcohol consumption* | 0.01 | 0.10 | 0.92 |
| Body adiposity [%] | -0.02 | -0.22 | 0.83 |

Table S10. Regression analysis for the relationship between TAC level and AR CAGn, testosterone (Model 1) or free testosterone (Model 2), and the interaction between the two, controlled for body adiposity (N = 131).

|  | β | t (126) | *p* |
| --- | --- | --- | --- |
| Model 1 | F(4,126) = 0.63, *adj. R^2^* = -0.01, *p* = 0.64 | | |
| AR CAGn | -0.03 | -0.34 | 0.73 |
| LOG T [ng/dl] | -0.13 | -1.30 | 0.20 |
| AR CAGn x T | 0.01 | 0.08 | 0.93 |
| Body adiposity [%] | 0.003 | 0.03 | 0.98 |
| Model 2 | F(4,125) = 2.33, *adj. R^2^* = -0.01, *p* = 0.55 | | |
| AR CAGn | -0.05 | -0.57 | 0.57 |
| LOG fT [ng/dl] | -0.08 | -0.88 | 0.38 |
| AR CAGn x fT | -0.11 | -1.08 | 0.28 |
| Body adiposity [%] | 0.02 | 0.23 | 0.82 |
